# Inhibition of heparanase protects against chronic kidney dysfunction following ischemia/reperfusion injury

**DOI:** 10.18632/oncotarget.26324

**Published:** 2018-11-16

**Authors:** Valentina Masola, Gloria Bellin, Gisella Vischini, Luigi Dall'Olmo, Simona Granata, Giovanni Gambaro, Antonio Lupo, Maurizio Onisto, Gianluigi Zaza

**Affiliations:** ^1^ Renal Unit, Department of Medicine, University Hospital of Verona, Verona, Italy; ^2^ University of Padova, Department of Biomedical Sciences, Padua, Italy; ^3^ Maria Cecilia Hospital, GVM Care and Research, Cotignola, Ravenna, Italy; ^4^ Università Cattolica del Sacro Cuore, Rome, Italy; ^5^ Azienda Ulss 3 Serenissima-Ospedale San Giovanni e Paolo, Venice, Italy

**Keywords:** heparanase, fibrosis, chronic kidney disease, inflammation

## Abstract

Renal ischemia/reperfusion (I/R) injury occurs in patients undergoing renal transplantation and with acute kidney injury and is responsible for the development of chronic allograft dysfunction as characterized by parenchymal alteration and fibrosis. Heparanase (HPSE), an endoglycosidase that regulates EMT and macrophage polarization, is an active player in the biological response triggered by ischemia/reperfusion (I/R) injury.

I/R was induced *in vivo* by clamping left renal artery for 30 min in wt C57BL/6J mice. Animals were daily treated and untreated with Roneparstat (an inhibitor of HPSE) and sacrificed after 8 weeks. HPSE, fibrosis, EMT-markers, inflammation and oxidative stress were evaluated by biomolecular and histological methodologies together with the evaluation of renal histology and measurement of renal function parameters.

8 weeks after I/R HPSE was upregulated both in renal parenchyma and plasma and tissue specimens showed clear evidence of renal injury and fibrosis. The inhibition of HPSE with Roneparstat-restored histology and fibrosis level comparable with that of control. I/R-injured mice showed a significant increase of EMT, inflammation and oxidative stress markers but they were significantly reduced by treatment with Roneparstat. Finally, the inhibition of HPSE *in vivo* almost restored renal function as measured by BUN, plasma creatinine and albuminuria.

The present study points out that HPSE is actively involved in the mechanisms that regulate the development of renal fibrosis arising in the transplanted organ as a consequence of ischemia/reperfusion damage. HPSE inhibition would therefore constitute a new pharmacological strategy to reduce acute kidney injury and to prevent the chronic pro-fibrotic damage induced by I/R.

## INTRODUCTION

Ischemia/reperfusion is defined as a clinical-pathological condition in which an initial limitation of blood supply to an organ occurs, followed by a subsequent phase of perfusion and re-oxygenation. Failure to supply arterial blood causes a serious imbalance in normal metabolic exchanges, causing tissue hypoxia. The subsequent restoration of blood flow and re-oxygenation is very often associated with further tissue lesions and a deep inflammatory response. Injuries caused by reperfusion aggravate a wide range of pathologies such as myocardial infarction, brain stroke, and acute kidney injury. The biological response induced by ischemia/reperfusion is various and includes cell death programs, vascular leakage, transcription reprogramming, activation of innate and adaptive immune cells, activation of complement and autoimmunity, partial epithelial-to-mesenchymal transition (EMT) and endothelial-to mesenchymal transition [1].

Renal ischemia/reperfusion (I/R) injury occurs in various clinical settings such as kidney transplantation, hemorrhagic shock, cardiovascular surgery, etc. [1, 2]. Although strategies have been tested and implemented to avoid or relieve renal I/R, the morbidity and mortality of the ensuing ischemic acute kidney injury (AKI) still remains high [3]. In transplanted patients it is associated with delayed graft function (DGF) and the development of chronic allograft nephropathy (CAN) [3, 4]. In settings other than kidney transplantation, patients who survive AKI can develop, in the long term, chronic kidney disease (CKD) and end-stage renal disease (ESRD).

CAN and CKD have been shown to be related to the development of tubule-interstitial fibrosis that alter the function and physiology of the organ [4]. Actually, the degree of tubulo-interstitial scarring is an excellent prognostic marker for ESRD [5]. The fibrotic process is sustained by the partial EMT of tubular cells [6]. This process is induced by hypoxia and reactive oxygen species (ROS) [7] together with the pro-fibrotic growth factors FGF −2 and TGF-β [8, 9].

During the EMT process, renal tubular cells activate transcription programs that result in loss of epithelial markers, upregulation of mesenchymal markers such as vimentin (VIM), α smooth muscle actin (α-SMA) and fibronectin (FN). As a consequence, these cells acquire a myofibroblast-like phenotype. In particular, in the partial EMT, tubular epithelial cells contribute to fibrosis production and express mesenchymal markers, without becoming motile or damaging epithelial structure [10, 11].

It has been widely demonstrated that heparanase (HPSE) plays an important role in regulating fibrosis and EMT [12–14]. HPSE is an endo-β-(1, 4)-D-glucuronidase that cleaves the heparan sulphate (HS) chains of HS-proteoglycans. It takes part in extracellular matrix remodeling and turnover as well as in releasing and facilitating the diffusion of HS-bound molecules (growth factors, cytokines). In normal tissue and in physiological conditions, HPSE is expressed at low levels. Whereas, in pathological conditions such as tumor progression and metastasis, inflammation and fibrosis, it is overexpressed [15].

Today the role of HPSE in the development of kidney disease, and its potential as a therapeutic target is well recognized [12, 16, 17]. In particular, HPSE alters glomerular structures, thereby facilitating the development of albuminuria and further glomerular inflammation [16]. Increased renal HPSE is also involved in EMT during acute kidney injury [12].

HPSE modulates tubular cells EMT in many different ways by regulating: the availability and activity of growth factors (i.e. FGF-2 and TGF-β) [13, 18], the infiltration and polarization of macrophages, the production of pro-inflammatory and pro-fibrotic cytokines, and the cross-talk macrophages/tubular cells shortly after I/R [19].

We have recently demonstrated in a murine model that HPSE is involved in acute renal damage after I/R. In fact, its inhibition prevented inflammation and tubular injury [19]. Whether HPSE also has a role in the chronic renal damage and fibrosis induced by I/R and whether HPSE inhibition could prevent it is not known. The results of the follow-up study after 8 weeks from the I/R insult of the referred experiment [19] are reported in this paper.

## RESULTS

### Up-regulation of HPSE 8 week after I/R

**Figure 1:**
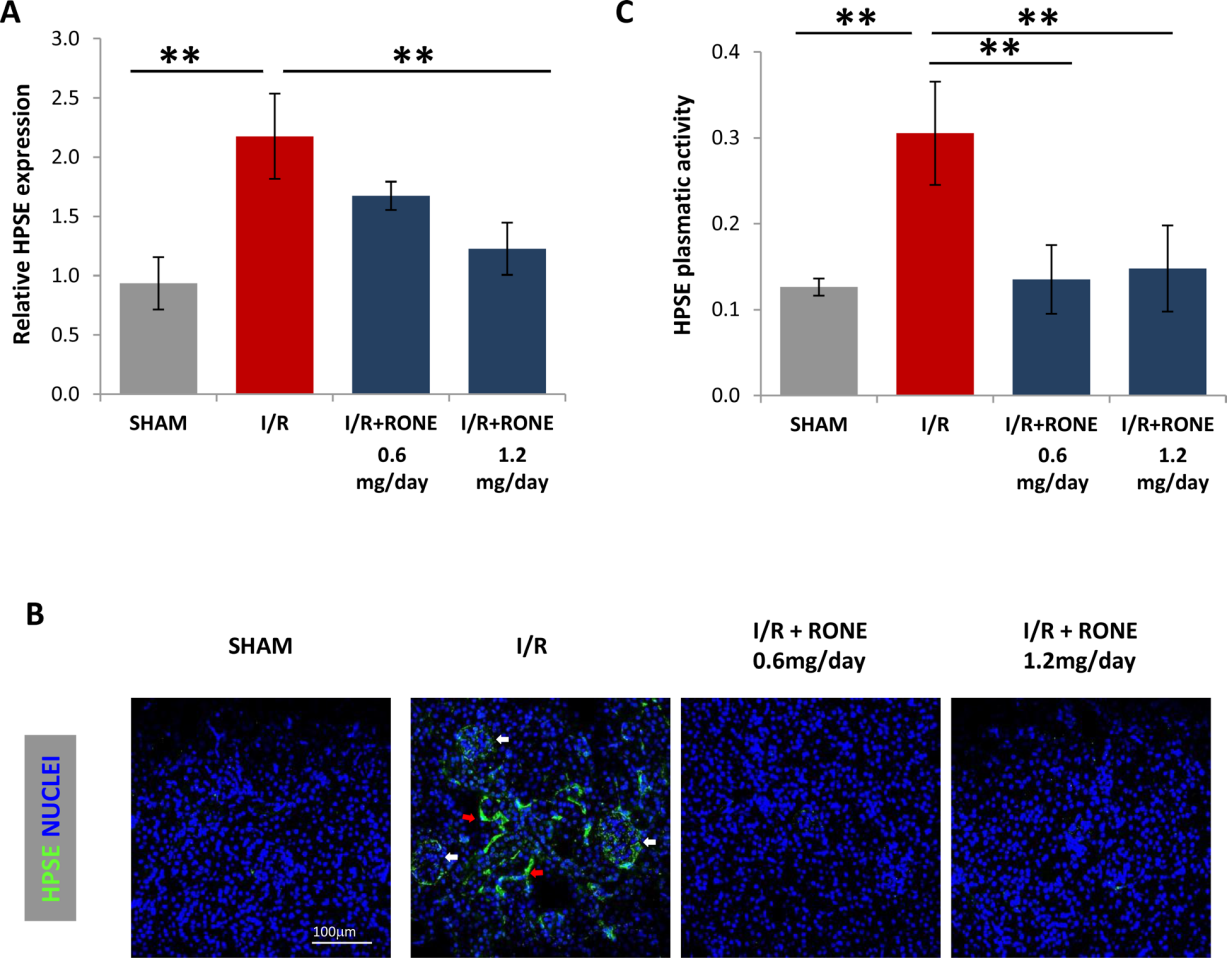
HPSE expression and activity induced by I/R (**A**) Histogram showing relative gene expression of HPSE evaluated by real-time PCR in renal tissue (*N* = 7). Results were normalized to GAPDH expression. (**B**) Representative immunofluorescence staining for HPSE (green) in cortical renal tissues. Nuclei were counterstained in blue. White arrows indicate HPSE expression in glomeruli; red arrows indicate HPSE expression in interstitial cells. (**C**) Histogram showing HPSE activity evaluated by ELISA in plasma samples collected from killed mice. ^**^P < 0.001

Eight weeks after the operation, gene expression analysis of the total renal parenchyma revealed the significant up-regulation of HPSE in I/R-injured mice while it was significantly reduced by treatment with RONE (Figure 1A). Tissue HPSE protein was also increased after I/R, and again the effect was partially blocked by the administration of RONE. Immunofluorescence staining demonstrated that HPSE was expressed in the glomeruli and in the interstitial cells (Figure 1B). Correspondingly, HPSE plasma levels were significantly increased in I/R-injured mice and the treatment with both RONE doses decreased concentration to basal levels (Figure 1C).

### RONE attenuates chronic morphological changes and fibrosis after I/R

**Figure 2:**
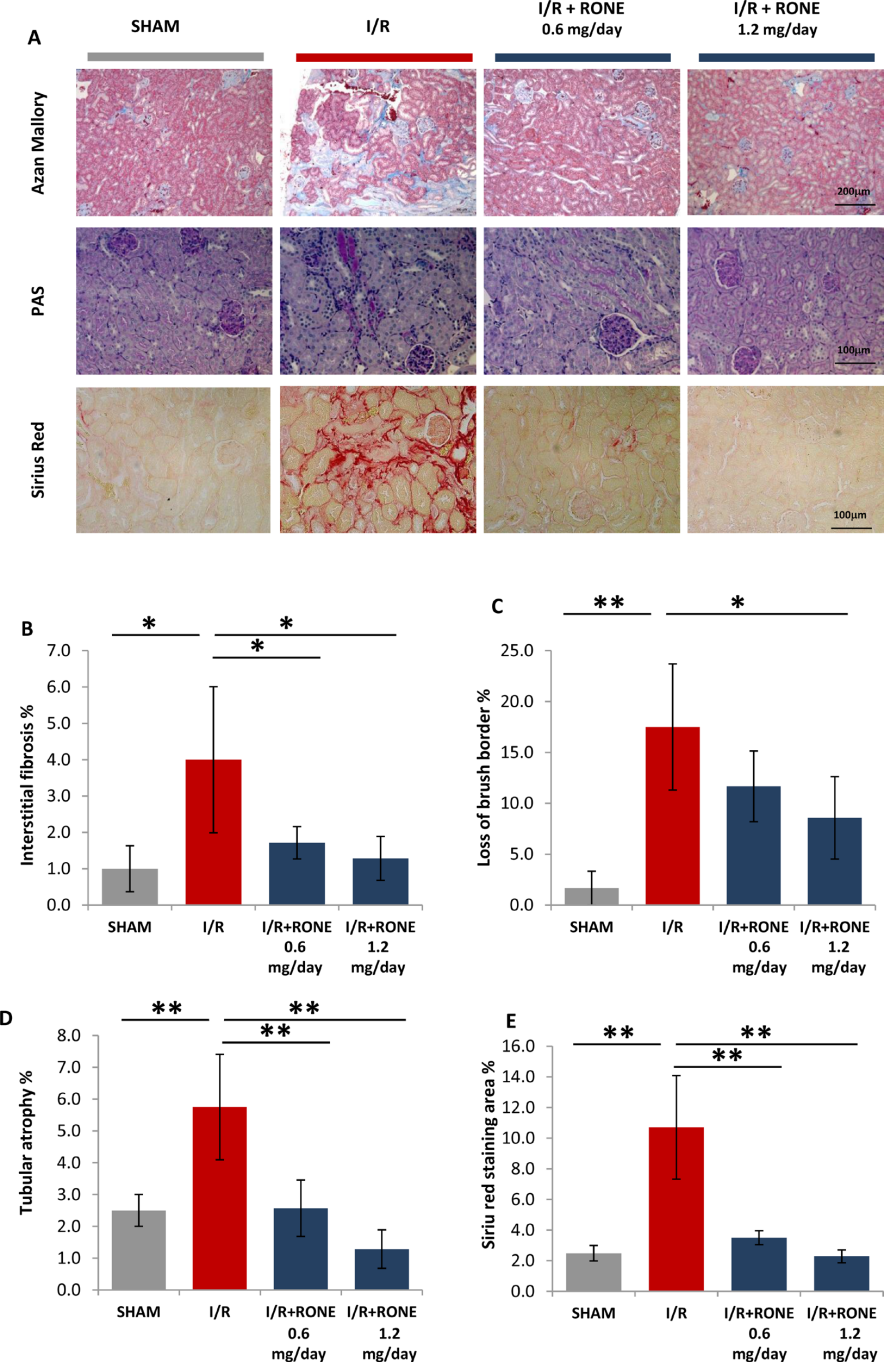
HPSE inhibition ameliorates renal injury and interstitial fibrosis induced I/R (**A**) Representative light microscopy images of Azan-Mallory, PAS and Siriur Red stained sections of the renal cortex from each group (scale bar = 200 μm). Histograms represent quantification of renal injury: (**B**) interstitial fibrosis (**C**) loss of brush border and (**D**) tubular atrophy (blubbing and sloughing of epithelial tubular cells) evaluated by a skilled pathologist in a blinded manner. Value are expressed as percentage of the observed area. (**E**) Histogram represent quantification of Sirius Red positive area. Values are expressed as mean ± standard deviation; *N* = 7. ^**^p < 0.001; ^*^p < 0.05.

To visualize fibrosis in renal tissue, Azan-mallory stain was performed. This staining allows evaluation of localization and severity of deposition of the extracellular matrix colored in blue. In I/R mice, we found prominent fibrosis in the interstitial cortex which was significantly reduced by treatment with both RONE doses (Figure 2A and 2B).

To evaluate the extent of chronic kidney injury, renal sections were stained with PAS. As expected, PAS staining proved that I/R induced tubular injury and severe loss of structure as shown by the loss of brush border, detachment from the basement membrane, bubbling and sloughing of tubular cells and the formation of intratubular casts. These events were substantially reduced in I/R mice treated with RONE (Figure 2A, 2C, 2D). Quantification of collagen deposition by Sirius-red staining demonstrated an increased accumulation in I/R injured mice which resulted as being substantially abrogated in RONE treated mice (Figure 2A and 2E).

### HPSE inhibition ameliorated renal function in I/R induced chronic renal disease

**Figure 3:**
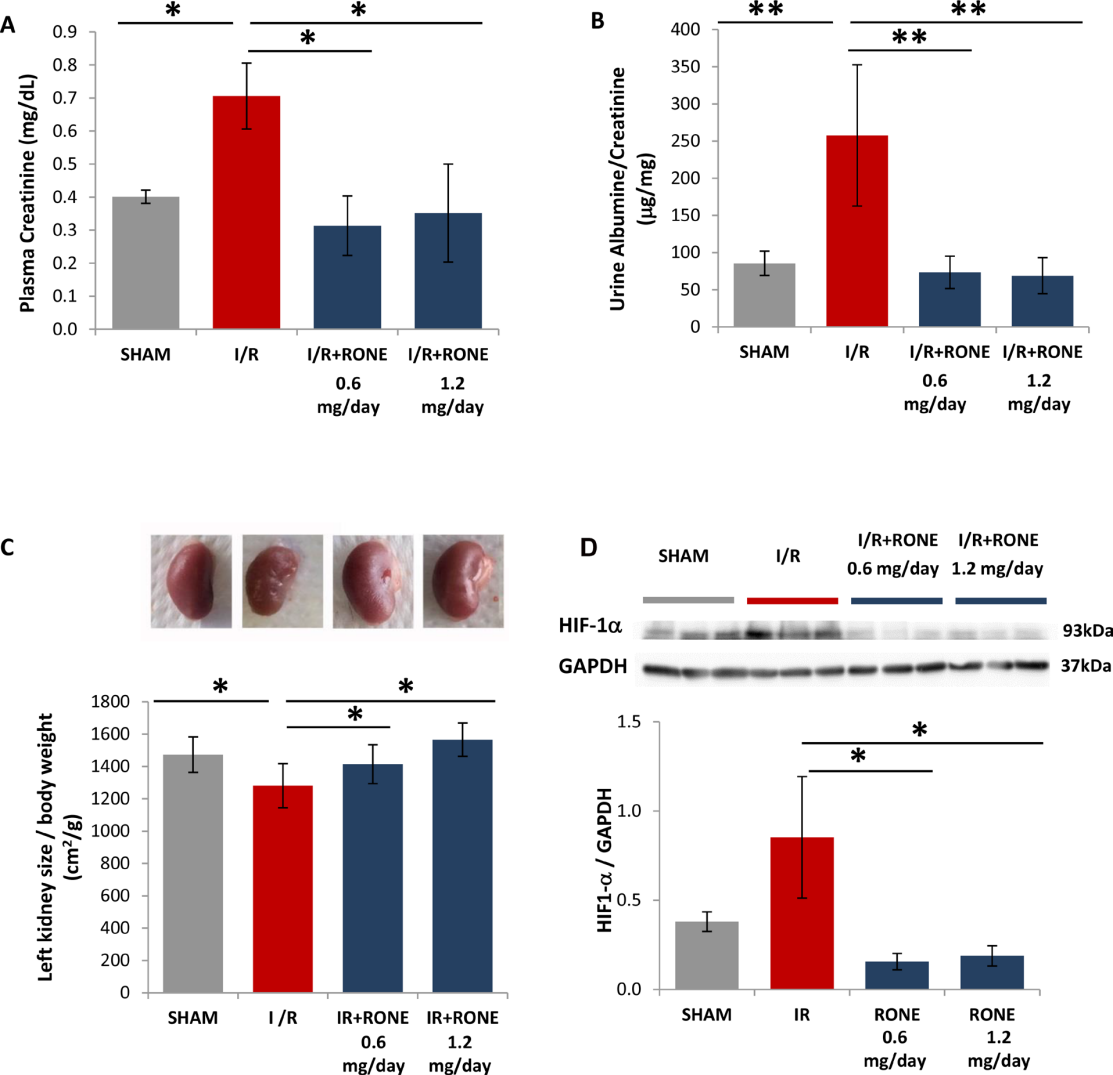
Biomarkers of renal function and kidney mass in RONEPARSTAT-treated and untreated mice subjected to I/R Effects of I/R on (**A**) Plasma Creatinine after I/R was measured in plasma collected at sacrifices from RONEPARSTAT-treated and untreated mice. (**B**) Urine Albumine/Creatinine ratios were measured in 24 hours urine collected the day previously the sacrifice. (**C**) *Upper:* Exemplificative images of left kidneys of the different mice groups. *Lower:* kidney size was calculated as ratio between left planar surface area and body weight. Results are expressed as mean ± SD.^**^p < 0.001; ^*^p < 0.05. (**D**) *Upper:* HIF1-α protein levels measured by Western blot analysis in randomly selected samples of total kidney lysates. GAPDH was used as loading control. *Lower:* Histogram represents its quantification normalized to GAPDH. ^*^p < 0.05.

As shown in Figure 3A, I/R-injured mice displayed an impaired renal function as indicated by remarkably increased plasmatic levels of creatinine compared with sham mice. Similarly, urinary albumin/creatinine ratios were also significantly increased in I/R-injured mice (Figure 3B). On the contrary, all parameters were reduced to basal level in I/R-injured mice treated with RONE (Figure 3A and 3B).

The kidney that underwent I/R was significantly reduced in volume compared with the contralateral which appeared clearly shrunken. In contrast, mice treated with both doses of RONE displayed a normal renal mass and surface aspect (Figure 3C).

Loss of functional mass after renal ischemia-reperfusion injury is supposed due to chronic oxygen deprivation to the tubulo-interstitial compartment, caused by the damage to the post-glomerular capillary circulation. As shown in Figure 3D, HIF1-α was significantly up-regulated in I/R-injured mice, whereas its protein amount is comparable with SHAM mice in I/R-injured mice treated with RONE.

### RONE reduces the expression of EMT and fibrosis associated markers after I/R

**Figure 4:**
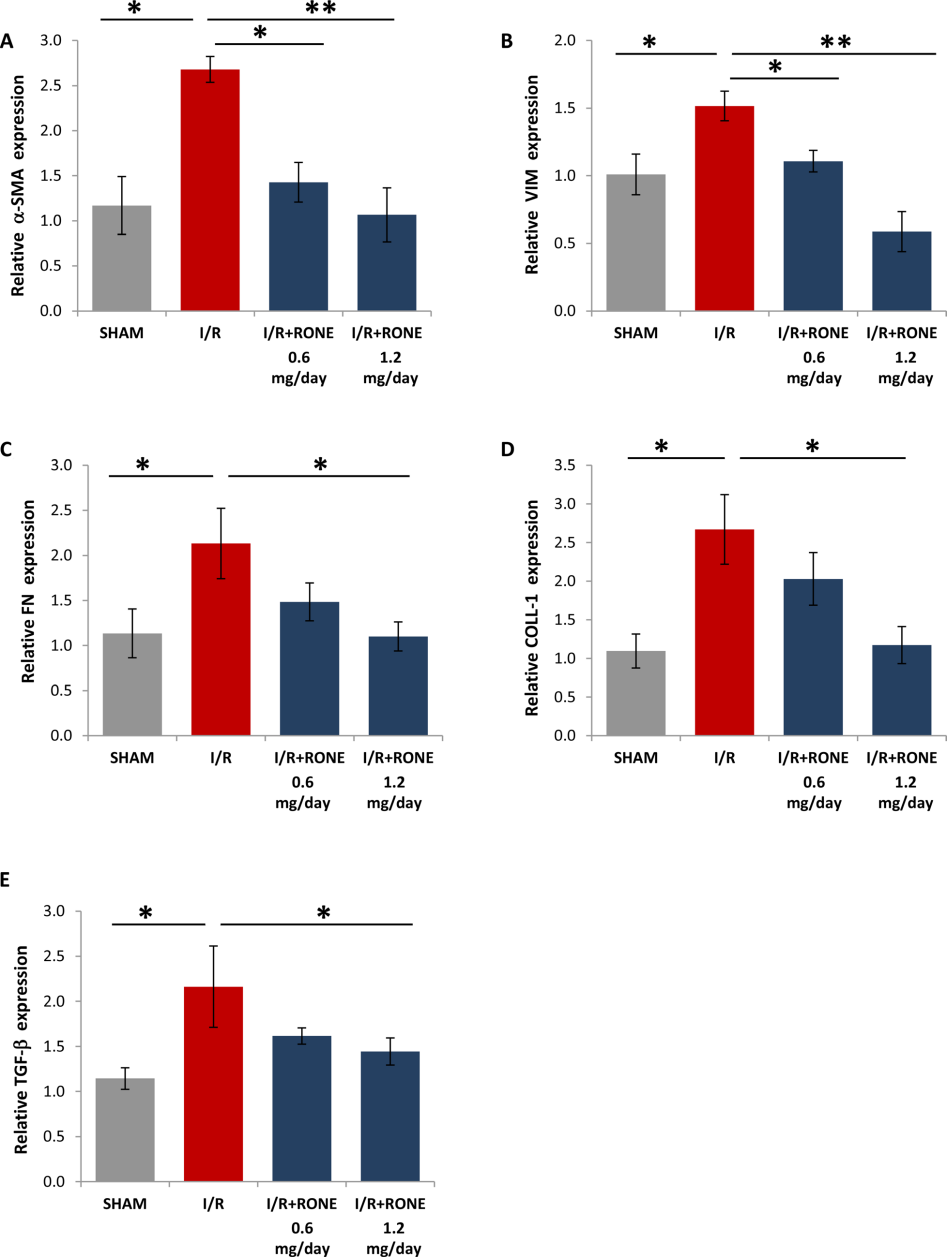
Gene expression of fibrotic markers Relative gene expression of (**A**) α-SMA, (**B**) VIM, (**C**) FN, (**D**) COLL-1 and (**E**) TGF-b was evaluated by real-time PCR in renal tissue extracts from mice subjected to I/R kidney injury and treated or untreated with RONEPARSTAT. Results were normalized to GAPDH expression. Histograms represent mean ±S.D of two separate experiments performed in triplicate.^**^p < 0.001; ^*^p < 0.05.

**Figure 5:**
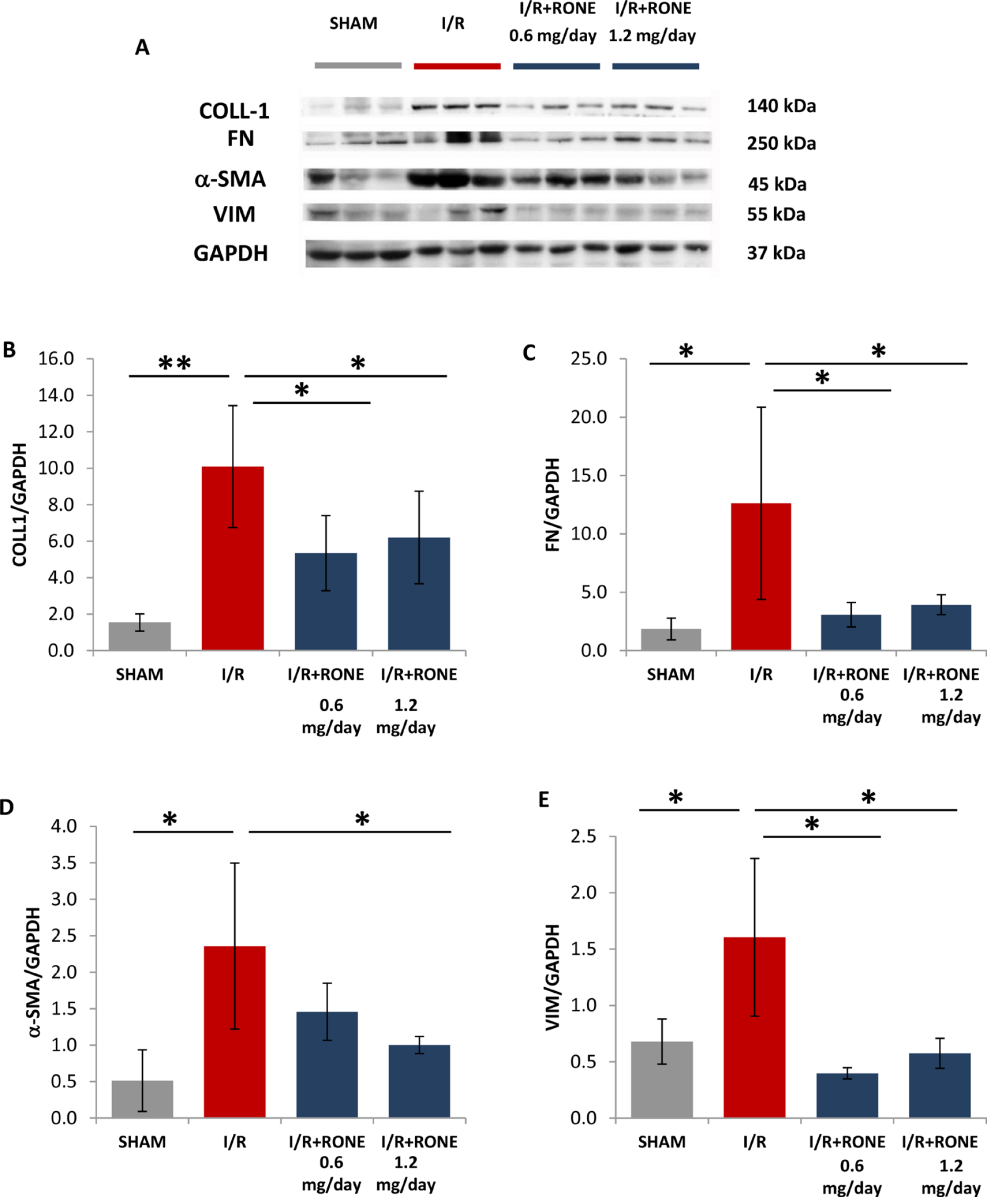
Protein expression of fibrotic markers (**A**) Collagen-1, Fibronectin, a-SMA and Vimentin protein levels measured by Western blot analysis in randomly selected samples of total kidney lysates. GAPDH was used as loading control. Histograms, (**B**–**E**) represent their quantification normalized to GAPDH. ^**^p < 0.001; ^*^p < 0.05.

To assess whether HPSE was able to modulate the activation of partial EMT program in the chronic model of I/R injury, we evaluated gene and protein expression of several markers. Gene (Figure 4) and protein (Figure 5) expression analysis of total kidney lysates confirmed that 8 weeks after I/R mice the expression of α-SMA, VIM, FN, Coll-1 and TGF-beta were significantly upregulated and the treatment with both doses of RONE reported almost value to basal level.

**Figure 6:**
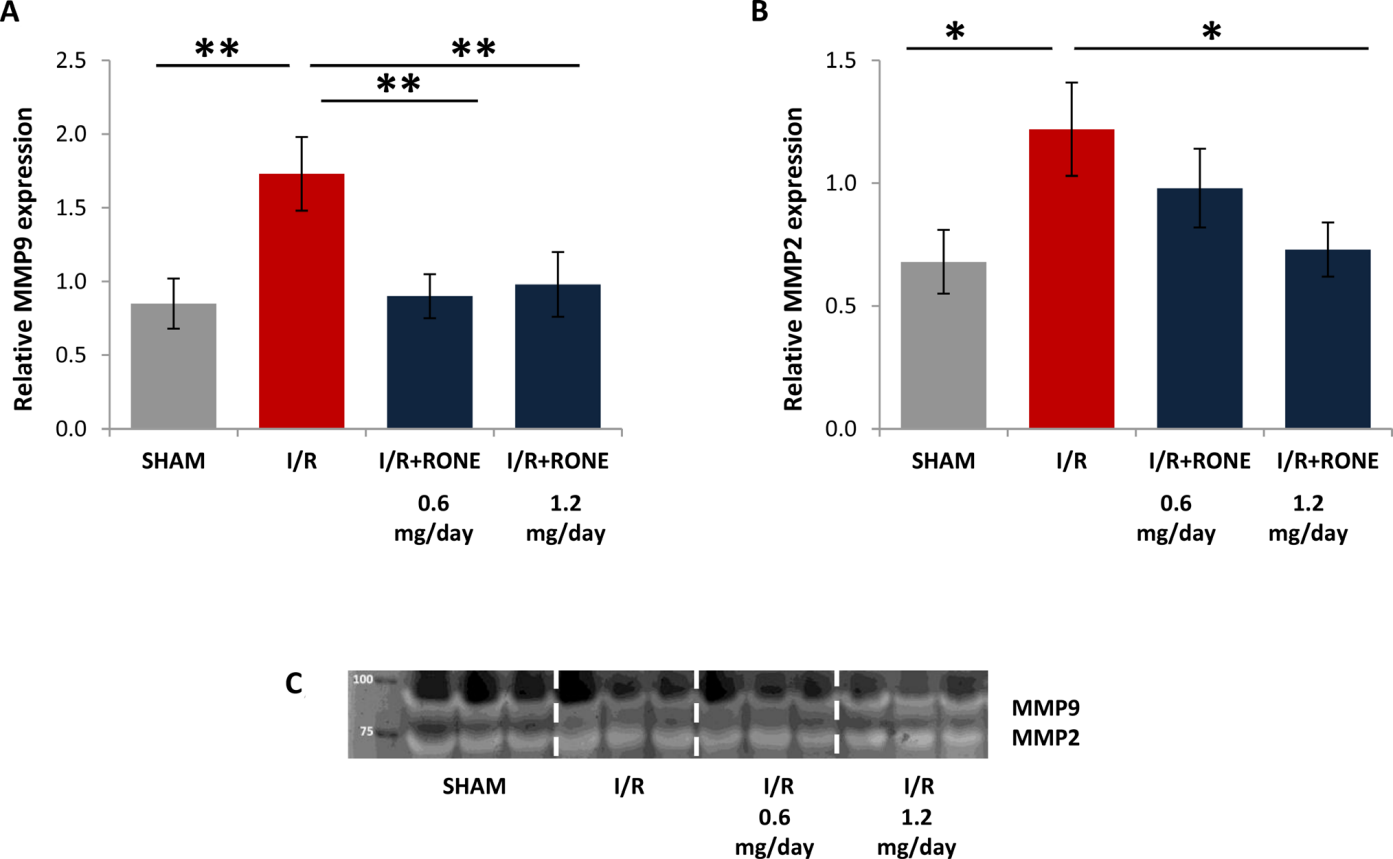
Gene expression of MMPs Relative gene expression of (**A**) MMP-9 and (**B**) MMP-2 was evaluated by real-time PCR in renal tissue extracts from mice subjected to I/R kidney injury and treated or untreated with RONEPARSTAT. Results were normalized to GAPDH expression. Histograms represent mean ± S.D of two separate experiments performed in triplicate.^**^p < 0.001; ^*^p < 0.05. (**C**) Gelatin zymography shows MMP-9 and MMP-2 activity bands in the renal tissue of the different groups of mice.

Matrix metalloproteinases (MMPs), in particular MMP9 and MMP2, are up-regulated in chronic kidney diseases and they sustain fibrosis via TGF-beta pathway [20]. Since HPSE modulate MMP expression [21] we assessed MMP-9 and MMP-2 expression and activity in renal tissue. I/R injury resulted in gene up-regulation of these MMPs and the treatment with both doses of RONE reduced their expression (Figure 6A and 6B). Differentially, MMPs activity is similar in all the groups (Figure 6C).

### HPSE inhibition reduces inflammation and chronic oxidative stress

**Figure 7:**
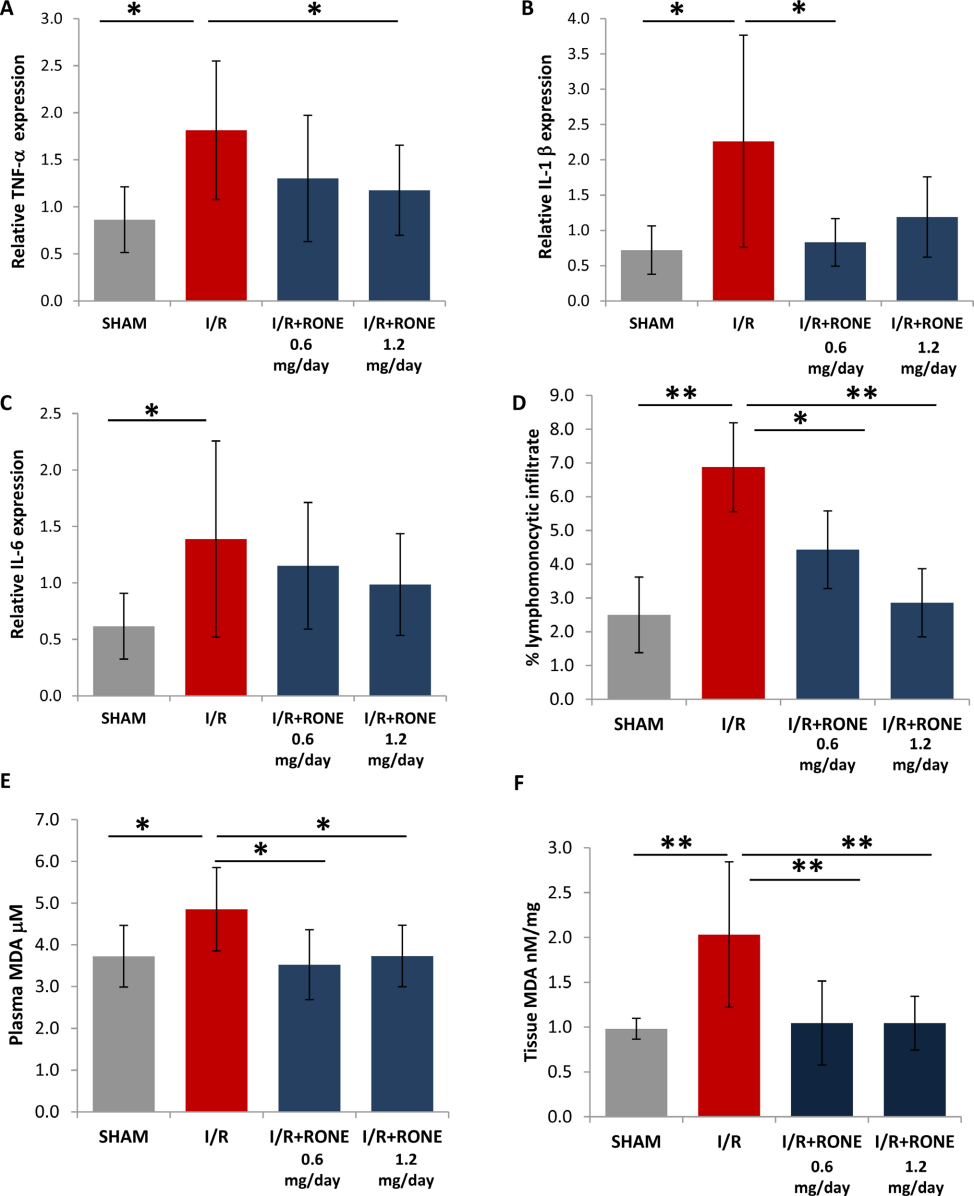
Regulation of inflammation and oxidative stress Relative gene expression of (**A**) TNF-α, (**B**) IL-1b and (**C**) IL-6 was evaluated by real-time PCR in renal tissue extracts from mice subjected to I/R kidney injury and treated or untreated with RONEPARSTAT. Results were normalized to GAPDH expression. (**D**) Lymphomonocytic infiltration was evaluated by a skilled pathologist in a blinded manner on histological slides. Histogram represent percentage of the observed area and value are expressed as mean ± standard deviation; *N* = 7. ^**^p < 0.001; ^*^p < 0.05. Malondialdehyde (MDA) was measured as TBARS in (**E**) plasma and (**F**) renal tissue. Histograms represent mean ± S.D of two separate experiments performed in triplicate. ^**^p < 0.001; ^*^p < 0.05.

Since it is known that cytokines modulate the interaction between immune and renal parenchymal cells to mediate kidney injury and fibrosis [19], we measured the expression of pro-inflammatory cytokines (TNF-α, IL-1β and IL-6) in renal tissue to assess the role of HPSE on the regulation of chronic inflammation. As expected, I/R-injured mice showed a significant increase of TNF-α, IL-1b and IL-6 gene expression [22]. HPSE inhibition with RONE significantly reduced TNF-α and IL-1β expression and partially lowered IL-6 expression (Figure 7A–7C). Histological evaluation observed a significant lymphomonocyte infiltration in renal parenchyma of I/R-injured mice which was reduced with the RONE treatment (Figure 7D).

To assess the role of HPSE on the regulation of oxidative stress, we assayed the plasmatic and renal tissue amount of Thiobarbituric Acid Reactive Substances (TBARS), products of oxidative damage to lipid.

In particular, we analyzed the levels of malondialdehyde (MDA), one of several low-molecular-weight end products formed via the decomposition of certain primary and secondary lipid peroxidation products. Results showed that mice subjected to I/R were characterized by higher TBARS value both in plasma and renal tissue with respect to sham animal. Whereas, in RONE-treated mice, TBARS levels were comparable with control mice (Figure 7E and 7F).

### HPSE inhibition regulates Nitric Oxide and Endothelin systems

Since Endotheli-1 (ET-1) and nitric oxide systems are important regulators of renal fibrosis [23, 24] we measured ET-1, endothelin receptor A (EDNRA), endothelial nitric oxide synthase (eNOS) and inducible nitric oxide synthase (iNOS) gene expression by real-time PCR.

**Figure 8:**
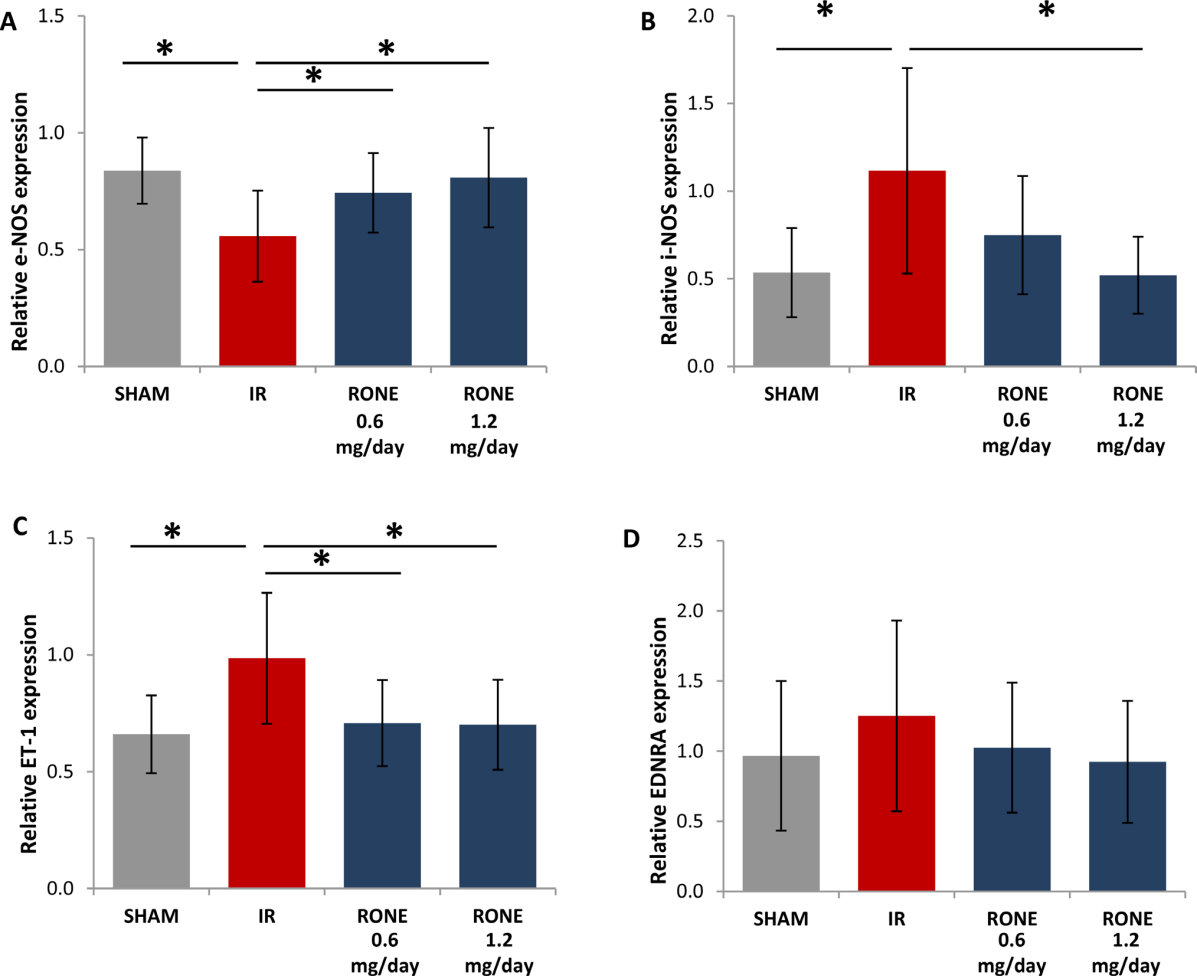
Regulation of endothelin and Nitric Oxide systems Relative gene expression of (**A**) eNOS, (**B**) iNOS, (**C**) ET-1 and (**D**) EDNRA was evaluated by real-time PCR in renal tissue extracts from mice subjected to I/R kidney injury and treated or untreated with RONEPARSTAT. Results were normalized to GAPDH expression. Histograms represent mean ± S.D of two separate experiments performed in triplicate.^**^p < 0.001; ^*^p < 0.05.

Results proved that I/R-injured mice have reduced eNOS and increased iNOS and ET-1 expression. EDNRA was slightly increased in I/R-injured mice. Differentially, I/R-injured mice treated with RONE displayed eNOS, iNOS, ET-1 and EDNRA expression levels similar to sham group (Figure 8).

## DISCUSSION

Significant improvements in graft survival have been observed over the last few decades by improving kidney preservation, and with immunosuppressive and supportive therapies. However, graft survival has reached a plateau because the onset of CAN limits long-term graft survival [25]. CAN is the most common cause for late kidney graft loss in addition to death with functioning graft. The clinical course of CAN is characterized by the progressive dysfunction of the transplanted kidney as manifested by slowly increasing in serum creatinine, mounting proteinuria and worsening hypertension [2]. Both alloantigen-dependent and -independent processes [3, 26] take part in the development of CAN [27]. Among the latter processes, ischemia/reperfusion and delayed graft function due to ischemic renal damage have been associated with CAN [1, 4]. Thus, measures to prevent the renal damage induced by I/R could be valuable in decreasing the incidence of CAN and prolonging graft survival.

HPSE is engaged in different renal pathological disorders [16]. We have recently demonstrated in cell cultures and in a murine model that HPSE is involved in acute renal damage after I/R [19, 28]. In particular, HPSE regulates the M1 polarization of macrophages induced by H/R-injured tubular cells and the early activation of partial epithelial–mesenchymal transition of these epithelial cells induced by M1 macrophages. These effects are prevented by the inhibition of HPSE [19, 28]. Since M1 macrophages and EMT are known factors in the chronicization of acute renal injury, we hypothesized that HPSE inhibition prevents the chronic renal damage induced by I/R.

In the present study, we show that I/R induces the chronic renal over-expression of HPSE after the initial insults. In particular, we detected HPSE overexpression after 8 weeks from the I/R insult both in glomeruli and interstitial cells (fibroblasts and cells of the immune system). Results in the RONE-treated animals confirm our hypothesis.

Chronic renal damage after I/R injury is characterized by glomerulosclerosis, tubular interstitial fibrosis, and tubular atrophy [29] associated with loss of microvasculature [30]. In humans, patients that survived up to 5 years after transplant, a predominant finding on protocol biopsies is interstitial fibrosis/tubular atrophy [31], a finding that we also observed in I/R-injured chronic mice. They had in fact tubular casts and debris, atrophic tubuli, tubulo-interstitial fibrosis characterized by collagen I deposition. HPSE inhibition by RONE completely abrogated the I/R induced chronic renal damage and restored normal kidney histology. In particular, HPSE inhibition completely prevented the development of fibrosis.

Loss of microvasculature, a typical finding in chronic interstitial renal damage [30], by causing chronic oxygen deprivation to the tubule-interstitial compartment, is one of the causes of loss of functional mass after renal I/R [28]. We indeed observed that macroscopically, I/R-injured chronic kidneys had a reduction in size and a shrunken appearance. The inhibition of this event, by HPSE-inhibition, is possibly also due to HPSE activity on HIF1-α [12, 32] a master regulator of chronic hypoxia [33]. Here we confirmed that HIF1-a is up-regulated in the I/R-injured mice but not in I/R-injured mice treated with RONE.

Chronic I/R-induced injury characterized by glomerular and tubular alterations is associated with renal functional impairment characterized by proteinuria. In fact, we observed a significant increase in plasma creatinine and albuminuria. Interestingly, a significant improvement of renal function was observed in RONE-treated I/R-injured chronic mice in parallel with the histological restoration.

In renal fibrosis, EMT was originally proposed to be the main source of activated myofibroblasts [34]. However, subsequent studies proved that tubular cells can also acquire mesenchymal traits, although still maintaining epithelial markers [35]. The acquisition of partial-EMT features however has a dramatic effect on tubular epithelial cells functions: the expression of fundamental proteins for absorption and secretion is reduced [36], G2-cell cycle arrest is induced, reducing the repair capacity of the damaged cells [37], and the epithelial secretome is altered fueling inflammation [38]. In our *in vivo* model, we confirmed that I/R-induced chronic injury up-regulates gene and protein expression of EMT-associated markers α-SMA, VIM, FN, COLL-I and the pro-fibrotic growth factor TGF-β. Remarkably, as reported in other settings [12], HPSE inhibition reduces the activation of EMT program with the consequent attenuation of fibrosis.

MMPs are crucial effectors in the development and progression of renal fibrosis. They are induced by hypoxia, TGF-β and they sustain the partial-EMT process of tubular cells in the early stages of CKD. In the late fibrotic stages, even if MMP expression is up-regulated, their activity is reduced due to the enhanced endocytosis, which is caused by hypoxia [20]. In our chronic renal fibrosis model we confirmed an increased expression of MMP9 and MMP2 but they activity in renal parenchyma was comparable to SHAM subject according to the present literature. Interestingly, HPSE inhibition resulted in a reduction of MMPs gene expression in I/R-injured mice, as previously described in mouse and cellular models [21, 13]. Even if MMPs activity is not affected, the reduction of MMPs expression by RONE could be equally important since MMPs also exert non-proteolytic activities, such as the activation of PI3K/AKT and ERK pathway [39, 40] which can sustain the progression of fibrosis.

However, the reno-protective effects of RONE on I/R-induced chronic damage and fibrosis appears to be more complex than just the inhibition of EMT, there also being evident an effect of this drug on chronic inflammation and oxidative stress. Similarly to what has been observed in CKD patients [41], we also observed increased HPSE activity in plasma of I/R-injured mice. We speculate that in this situation HPSE may fuel a systemic chronic pro-inflammatory state. Here we prove that treatment with RONE not only completely inhibits HPSE activity but also reduces its renal expression.

Several pieces of evidence indicate that renal inflammation plays a central role in the initiation and progression of CKD and in the regulation of fibrosis progression [42]. In particular, renal I/R injury leads to recruitment and activation of inflammatory cells both in the short and long term [20, 43]. HPSE is an important regulator of inflammation in several settings [44] including acute I/R injury [19]. Our findings confirm that the inflammatory cytokines TNF-α, IL-1β and IL-6 remain increased in the renal tissue well after I/R and that the inhibition of HPSE results in a reduction of their expression levels and consequently of inflammation.

It has been reported that during renal I/R injury, oxidative stress is one of the most critical mechanisms involved in tubular cellular damage [45]. Oxidative stress is known to increase as chronic kidney disease progresses and correlates significantly with the level of renal function [46, 47]. Here we show that I/R chronically injured mice display a significant increase in serum and renal tissue MDA levels which interestingly are maintained in the normal range by RONE treatment [48].

Nitric oxide signal transduction plays an important role in renal ischemia/reperfusion (I/R) injury. NO produced by eNOS has protective functions, whereas NO from iNOS induces impairment. Cellular effects of NO depend on its concentration, site of release and duration of action [49]. NO produced by eNOS is transient and its inactivation/expression reduction is a hallmark of endothelial dysfunction and consecutive vasoconstriction [50–52]. On the other hand when iNOS is up-regulated in response to pro-inflammatory and ischemic stimuli, it generates 100–1,000 fold more NO than does eNOS [53, 54] It has been hypothesized that this prolonged and high-output NO production leads to the generation of peroxynitrite anion which sustain lipid peroxidation, DNA damage and apoptosis and also to the down activation of eNOS creating a vicious loop [53]. In the present study, we confirmed a reduction of eNOS and an increase of iNOS in renal tissue of I/R-injured mice. The effects were blunted by the RONE treatment. Our findings thus confirm a role of HPSE in the regulation of eNOS/iNOS balance. Previously, studies proved that HS degradation of endothelial glycocalyx impairs the ability of endothelial cells to sense shear stress and to up-regulate eNOS [55]. Moreover, eNOS prevents heparanase induction and the development of proteinuria in a model of focal segmental glomerulosclerosis [56]. We could speculate that HPSE inhibition by RONE could preserve endothelial glycocalyx integrity and its eNOS expression which subsequently could help in the reduction of HPSE expression [57]. In line with what has been observed in a model of acute I/R injury [19], here, we proved that HPSE inhibition reduces iNOS production and likely NO abundance and the consequents effects. Another two elements that are reported to be involved in the development of acute and chronic renal disease are ET-1 and its receptor A [58]. ET-1 is a protein primarily produced by endothelial cells but in the kidneys, in nephrotic states, ET-1 may also be produced by tubular epithelial cells [59]. ET-1 is a potent vasoconstrictor by the interaction with its receptor A [60]. This, subsequently, increases glomerular pressure and renal ischemia than can, in turn, induce renal fibrosis [23]. ET-1 also promotes extra-cellular matrix protein synthesis through increased inflammation, reactive oxygen species and activation of the renin angiotensin system [58, 60]. In particular I/R injury up-regulates the expression of ET-1 and its receptor A [61]. Here we confirmed that chronic fibrosis activated by I/R injury is characterized by a significant ET-1 up-regulation. The expression of receptor A was only slightly increased. As previously described [62], ET-1 could participate in the increased HPSE production in I/R-injured fibrotic mice. Interestingly, in our *in vivo* model, HPSE inhibition reduced ET-1 up-regulation. The promoter of the EDN1 gene presents numerous regulatory elements and its transcription is therefore regulated by many hormonal and environmental stimuli (tumor necrosis factor-alpha, interleukins, insulin, norepinephrine, angiotensin II, thrombin, natriuretic peptides, NO, prostacyclin) [63] and thus the amelioration of renal fibrotic state by RONE treatment could also be indirectly responsible for ET-1 expression reduction.

A limitation of this study is that we cannot disentangle the effect on the chronic renal damage of the acute amelioration of the I/R renal damage by HPSE inhibition [19] from the sustained 8-week inhibition. Although studies with a different design or on different models of chronic nephropathy are warranted to clarify this issue, we think that the overexpression of HPSE in the chronic damage induced by I/R suggests that HPSE chronic inhibition contributes anyway to the long-term favorable effect. A further limitation is that we assumed that all the effects observed with RONE treatment are explained by HPSE inhibition. While this is likely the case for most of them due to the pivotal role of HPSE in many of the biological cascades activated by I/R, and because of the highly selective and specific activity of RONE on HPSE, we cannot rule out that RONE may have a role in inflammation and oxidative stress or regulation of endothelin and nitric oxide systems by itself. RONE is a modified non-anticoagulant heparin that due to the fundamental similarities in structure with HS may have the capacity of interfering with the role of HS in inflammation, oxidative stress and the functions of endothelial glycocalyx [57, 64, 65].

In conclusion, HPSE inhibition with RONE protects the kidney from chronic damage and fibrosis induced by I/R. In particular, this drug reduces tubular atrophy and interstitial fibrosis TA/IF, ameliorates renal function, reduces the partial-EMT program, inflammation and oxidative stress. Interestingly, the doses capable of protecting the kidney are comparable to those proven active in several animal models [66] as well as those tested in a phase I study in humans and yielding measurable drug exposure without serious adverse events [67]. These findings suggest that RONE administration could effectively prevent CAN possibly improving clinical outcomes in transplanted patients. In addition, since HPSE is also involved in the development of fibrosis in different organs such as the liver, lungs and mesothelium [14, 68, 69], the use of RONE could represent an additional pharmacological weapon to progressively fight incurable morbidities such as Chronic Obstructive Pulmonary Disease or peritoneal fibrosis in peritoneal-dialyzed patients.

## MATERIALS AND METHODS

### Reagents

Roneparstat (RONE)(Leadiant Biosciences SA, Switzerland SA), Periodic acid–Schiff (PAS), Azan-Mallory and Sirius Red staining reagents, protease inhibitor cocktail -cOmplete™, heparin, primers (Sigma-Aldrich), Matrigel (BD Matrigel™ Basement Membrane Matrix - BD Biosciences) TRIzol reagent, SuperScript II Reverse Transcriptase, Power SYBR Green Master Mix 2x (Thermo Fisher Scientific).

### Animal model of renal ischemia

Animals were handled and the experiment was performed as described in [19] to which the reader is referred for details. In short, 28 wild-type C57BL/6J mice weighing 23–30 g were randomly assigned to control group (*N* = 7), I/R group (*N* = 7) and two I/R+RONE groups (*N* = 14 each). Renal ischemia, without contralateral nephrectomy, was induced by clamping the left renal artery for 30 min. The clamp was then removed, the kidney was observed to confirm the return of blood flow, and the abdominal wall incision was sutured. Control mice underwent the same procedures without clamping. After the operation, I/R+RONE mice were injected intraperitoneally (IP) daily with two different doses 0.6 or 1.2 mg RONE dissolved in water for 8 weeks. Control and I/R mice were injected daily with the same volume of water. 24 h urine was collected in single animal metabolic cages prior to sacrifice. Urine was thereafter centrifuged at 4000 g for 10 min, and the supernatant removed and stored at −80°C. The mice were sacrificed after 8 weeks from the surgery by cervical dislocation. Mice were weighed and both kidneys photographed. Blood samples were collected for the subsequent analyses. One half of the left kidney was snap-frozen in liquid nitrogen and stored at −80°C and the other half was fixed in formalin, embedded in paraffin.

### Histology

Azan-Mallory, PAS and Sirius Red staining were performed using standard procedures on paraffin sections (4-μm) to analyze renal morphology. Histopathological scores were evaluated by a skilled pathologist in a blinded manner. Interstitial fibrosis was evaluated on Azan-Mallory-stained slides while loss of brush border and tubular atrophy were quantified on PAS-stained slides. Values are expressed as percentage of the observed area of 10 non-overlapping random cortex fields. To determine positive area percentage of Sirius red-stained slides, 10 random fields per section were chosen and photographed. Positive staining areas were quantified using Image J software.

### HPSE activity

HPSE activity was measured using a standardized enzyme-linked immunosorbent assay (ELISA) [41] in plasma obtained from blood collected in EDTA. This assay is based on the capacity of HPSE to degrade heparan-sulphate present in Matrigel. Briefly, 25 ml of 200 mg/ml Matrigel dissolved in cold PBS were used to coat 96-well plate at room temperature for 1.5 hours. Plasma samples were diluted by a ratio 1:4 in 1.0 M Sodium Acetate (pH 5), 0.1 mg/ml BSA, 0.01% Triton X-100, protease inhibitor cocktail in presence or absence of LMWH (Low Molecular Weight heparin) (50 μg/ml). Plate was washed once with PBT (PBS, 0.0% v/v Tween-20). Samples were loaded on plate and incubated overnight at 37°C. Plate was washed and blocked in PBT, 0.5%BSA, at room temperature for 2 hours. Plate was incubated with anti-HS antibody (HepSS-1) diluted 1:500 in blocking buffer, 1 h at room temperature. Plate was washed and incubated with the secondary HRP-conjugated antibody 1 h at room temperature. OD405 absorbance was read in an ELISA plate reader (Tecan) after the addition of 50 ml of 2,2-azino-bi-(3-ethylbenzthiazoline-6-sulphonic acid). HPSE activity was calculated as the difference between the OD405 value with or without 50 mg/ml heparin.

### Renal function parameters

Serum creatinine, urine albumin and creatinine levels were determined using commercially available kits (Abcam).

### Body weight and Kidney size

Body weight was monitored from the surgery to the end of the experimentation and kidney size was calculated as the ratio between the left kidney planar surface area and body weight at the time of sacrifice [70].

### Gene expression

Total RNA was extracted from frozen renal tissues using TRIzol reagent according to the manufacturer's instructions. RNA yield and purity were checked using a NanoDrop spectrophotometer (EuroClone), and total RNA from each sample was reverse transcribed into cDNA using SuperScript II Reverse Transcriptase. Real-time PCR was performed on an ABI Prism 7500 device using Power SYBR Green Master Mix 2× (Thermo Fisher Scientific). The comparative Ct method (ΔΔCt) was used to quantify gene expression, and the relative quantification was calculated as 2^−ΔΔCt^. The presence of nonspecific amplification products was excluded by melting curve analysis (primers are listed in Table 1). Data were normalized to GAPDH expression.

**Table 1:** Real-time PCR primers used for gene expression analysis

Gene	Forward	Reverse	Amplicon (bp)
GAPDH	GGCAAATTCAACGGCACAGT	GTCTCGCTCCTGGAAGATGG	84
HPSE	CAAGAACAGCACCTACTCAAG	AGCAGTAGTCAAGGAGAAGC	155
αSMA	TGCTGGACTCTGGAGATGGT	ACGAAGGAATAGCCACGCTC	148
VIM	TCCAGAGAGAGGAAGCCGAA	AAGGTCAAGACGTGCCAGAG	75
COLL1	GAGTGGAAGTGTGAGCGACA	GGTGAGTCTGCGGTTGGTAA	97
TGFβ	GTGTGGAGCAACATGTGGAACTCTA	CGCTGAATCGAAAGCCCTGTA	174
IL6	CTGCAAGAGACTTCCATCCAGTT	GAAGTAGGGAAGGCCGTGG	70
IL1β	TGTTTTCCTCCTTGCCTCT	TGCCTAATGTCCCCTTGA	100
TNFα	CATCTTCTCAAAATTCGAGTGACAA	TGGGAGTAGACAAGGTACAACCC	175
FN	QuantiTec Primer Assay (Quiagen)	Cat. No. QT00135758	
MMP9	CTTGAAGTCTCAGAAGGTGGATC	CGCCAGAAGTATTTGTCATGG	135
MMP2	TGCCATCCCTGATAACCTGGAT	CTCTTCAGACTTTGGTTCTCC	108
ET-1	TGCTGTTCGTGACTTTCC	TGTTGACCCAGATGATGTC	198
EDNRA	GCTGGTTCCCTCTTCACTTAAGC	TCATGGTTGCCAGGTTAATGC	129
eNOS	CTGGCAGCCCCAAGACCTA	GTGACATCGCCGCAGACAA	112
iNOS	CAGCTGGGCTGTACAAACCTT	CATTGGAAGTGAAGCGTTTCG	95

### Protein expression

Frozen renal tissue was lysed in RIPA buffer with cOmplete™ Protease Inhibitor Mixture. Briefly, equal amounts of proteins were mixed with reducing sample buffer and denatured for 10 minutes at 100°C. Protein samples were then resolved by 10% SDS PAGE and electro-transferred to nitrocellulose membranes. Nonspecific binding was blocked for 1 h at room temperature with 3% BSA in TBST buffer (50 mM Tris-HCl pH 7.4, 150 mM NaCl, 0.1% Tween 20). Membranes were exposed to primary antibodies overnight at 4°C and incubated with a secondary peroxidase-conjugated antibody for 1 h at room temperature (Table 2). Detection was performed using a chemiluminescence substrate with Alliance system (UVItec).

**Table 2:** List of antibodies used for Western blot (WB), ELISA and immunofluorescence (IF) staining experiments

	Application	Catalog Number	Brand
anti-HPSE	IF	SC-25826	Santa Cruz Biotechnology
anti-GAPDH	WB	sc-25778	Santa Cruz Biotechnology
anti-COLLAGEN 1	WB	TA309096	Origene
anti-FIBRONECTIN	WB	sc-9068	Santa Cruz Biotechnology
anti-VIMENTIN	WB	sc-7557	Santa Cruz Biotechnology
anti-α-SMA	WB	A-5228	Sigma
anti-HS antibody	ELISA	HepSS-1	Seikagaku
anti-HIF1α	WB	GT10211	Genetex
anti-rabbit A488	IF	A-11034	ThermoFisher
anti-rabbit HRP	WB	sc-2004	Santa Cruz Biotechnology
anti-mouse HRP	WB	sc-2005	Santa Cruz Biotechnology
anti-mouse IgM HRP	ELISA	sc-2973	Santa Cruz Biotechnology

### Zymography

Gelatin substrate zymography was used to assess MMP-9 and MMP-2 activity. Equal amounts of tissue extract were resolved in non-reducing sample buffer on 10% SDS-polyacrylamide gels co-polymerized with 0.1% gelatin. After electrophoresis, the gels were washed twice for 30 min in 2.5% Triton X-100 at room temperature to remove SDS, then equilibrated for 30 min in collagenase buffer (50 mM Tris, 200 mM NaCl, 5 mM CaCl2, and 0.02% Triton X-100, pH 7.4), and finally incubated overnight with fresh collagenase buffer at 37°C. After incubation, gels were stained in 0.1% Coomassie Brilliant Blue R-250, 30% MetOH/10% acetic acid for 1 h and destained in 30% MetOH/10% acetic acid.

### Immunofluorescence

After deparaffinization and antigen unmasking, renal sections were permeabilized with 0.2% Triton X-100 in PBS for 10 min at RT and blocked in PBS supplemented with 1% BSA and 0.1% Triton X-100 for 1 h at RT. Slices were incubated overnight at 4°C with anti-HPSE antibody prepared in PBS supplemented with 1% BSA and 0.3% Triton X-100. Goat anti-rabbit Alexa Fluor 488 was used as secondary antibody. Nuclei were counterstained with Hoechst for 20 min at RT. Images were acquired using a confocal microscope Leica TCS SP5.

### Thiobarbituric acid reactive substances (TBARS) assay

Measurement of Malondialdehyde (MDA) as TBARS was performed using a commercially available kit (AbNOVA Abnova, Walnut, CA) according to the manufacturer's instructions. In brief, 50 ml of sample (plasma or renal tissue extract) was added with 50 ml SDS solution and the vials were swirled to mix, then 2 ml of Color Reagent was added and the vials were boiled for one hour. Vials were transferred on ice for 10 minutes and then centrifuge 10 minutes at 1600 × g at 4°C. 150 μl from each vial were loaded in triplicate to a clear 96 well plate. Absorbance was measured at 530 nm. The values of MDA for each sample were calculated using a standard curve. MDA content in tissue extract was normalized to total protein amount.

### Statistical analysis

Means ± SD of the real-time PCR data were calculated by Rest2009 software. Differences were analyzed by linear regression models with groups (CTR, I/R, I/R + RONE 0. 6 mg/day and I/R + RONE 1.2 mg/day) as categorical variables. Bonferroni-corrected adjusted means and differences were computed using the control as the referent group. A Bonferroni corrected *P* value of < 0.05 was considered statistically significant.

## References

[1] Rovcanin B, Medic B, Kocic G, Cebovic T, Ristic M, Prostran M (2016). Molecular Dissection of Renal Ischemia-Reperfusion: Oxidative Stress and Cellular Events. Curr Med Chem.

[2] Koyama I, Bulkley GB, Williams GM, Im MJ (1985). The role of oxygen-free radicals in mediating the reperfusion injury of cold-preserved ischemic kidneys. Transplantation.

[3] Brown JR, Rezaee ME, Marshall EJ, Matheny ME (2016). Hospital Mortality in the United States following Acute Kidney Injury. BioMed Res Int.

[4] Hewitson TD (2009). Renal tubulointerstitial fibrosis: common but never simple. Am J Physiol Renal Physiol.

[5] Nath KA (1998). The tubulointerstitium in progressive renal disease. Kidney Int.

[6] Nieto MA, Huang RY, Jackson RA, Thiery JP (2016). EMT: 2016. Cell.

[7] Zhang S, Tan X, Chen Y, Zhang X (2017). Postconditioning protects renal fibrosis by attenuating oxidative stress-induced mitochondrial injury. Nephrol Dial Transplant.

[8] Zell S, Schmitt R, Witting S, Kreipe HH, Hussein K, Becker JU (2013). Hypoxia Induces Mesenchymal Gene Expression in Renal Tubular Epithelial Cells: An in vitro Model of Kidney Transplant Fibrosis. Nephron Extra.

[9] Kanasaki K, Taduri G, Koya D (2013). Diabetic nephropathy: the role of inflammation in fibroblast activation and kidney fibrosis. Front Endocrinol (Lausanne).

[10] Cruz-Solbes AS, Youker K (2017). Epithelial to Mesenchymal Transition (EMT) and Endothelial to Mesenchymal Transition (EndMT): Role and Implications in Kidney Fibrosis. Results Probl Cell Differ.

[11] Sun YB, Qu X, Caruana G, Li J (2016). The origin of renal fibroblasts/myofibroblasts and the signals that trigger fibrosis. Differentiation.

[12] Masola V, Zaza G, Gambaro G, Onisto M, Bellin G, Vischini G, Khamaysi I, Hassan A, Hamoud S, Nativ O, Heyman SN, Lupo A, Vlodavsky I (2016). Heparanase: A Potential New Factor Involved in the Renal Epithelial Mesenchymal Transition (EMT) Induced by Ischemia/Reperfusion (I/R) Injury. PLoS One.

[13] Masola V, Gambaro G, Tibaldi E, Brunati AM, Gastaldello A, D'Angelo A, Onisto M, Lupo A (2012). Heparanase and syndecan-1 interplay orchestrates fibroblast growth factor-2-induced epithelial-mesenchymal transition in renal tubular cells. J Biol Chem.

[14] Lv Q, Zeng J, He L (2016). The advancements of heparanase in fibrosis. Int J Mol Epidemiol Genet.

[15] Rivara S, Milazzo FM, Giannini G (2016). Heparanase: a rainbow pharmacological target associated to multiple pathologies including rare diseases. Future Med Chem.

[16] Rabelink TJ, van den Berg BM, Garsen M, Wang G, Elkin M, van der Vlag J (2017). Heparanase: roles in cell survival, extracellular matrix remodeling and the development of kidney disease. Nat Rev Nephrol.

[17] Ramani VC, Zhan F, He J, Barbieri P, Noseda A, Tricot G, Sanderson RD (2016). Targeting heparanase overcomes chemoresistance and diminishes relapse in myeloma. Oncotarget.

[18] Masola V, Zaza G, Secchi MF, Gambaro G, Lupo A, Onisto M (2014). Heparanase is a key player in renal fibrosis by regulating TGF-β expression and activity. Biochim Biophys Acta.

[19] Masola V, Zaza G, Bellin G, Dall'Olmo L, Granata S, Vischini G, Secchi MF, Lupo A, Gambaro G, Onisto M (2018). Heparanase regulates the M1 polarization of renal macrophages and their crosstalk with renal epithelial tubular cells after ischemia/reperfusion injury. FASEB J.

[20] Cheng Z, Limbu MH, Wang Z, Liu J, Liu L, Zhang X, Chen P, Liu B (2017). MMP-2 and 9 in Chronic Kidney Disease. Int J Mol Sci.

[21] Zcharia E, Jia J, Zhang X, Baraz L, Lindahl U, Peretz T, Vlodavsky I, Li JP (2009). Newly generated heparanase knock-out mice unravel co-regulation of heparanase and matrix metalloproteinases. PLoS One.

[22] Ascon M, Ascon DB, Liu M, Cheadle C, Sarkar C, Racusen L, Hassoun HT, Rabb H (2009). Renal ischemia-reperfusion leads to long-term infiltration of activated and effector-memory T lymphocytes. Kidney Int.

[23] François H, Chatziantoniou C (2018). Renal fibrosis: Recent translational aspects. Matrix Biol.

[24] Eberhardt W, Pfeilschifter J (2007). Nitric oxide and vascular remodeling: spotlight on the kidney. Kidney Int Suppl.

[25] Chapman JR, O'Connell PJ, Nankivell BJ (2005). Chronic renal allograft dysfunction. J Am Soc Nephrol.

[26] Chawla LS, Eggers PW, Star RA, Kimmel PL (2014). Acute kidney injury and chronic kidney disease as interconnected syndromes. N Engl J Med.

[27] Ishani A, Xue JL, Himmelfarb J, Eggers PW, Kimmel PL, Molitoris BA, Collins AJ (2009). Acute kidney injury increases risk of ESRD among elderly. J Am Soc Nephrol.

[28] Abassi Z, Hamoud S, Hassan A, Khamaysi I, Nativ O, Heyman SN, Muhammad RS, Ilan N, Singh P, Hammond E, Zaza G, Lupo A, Onisto M (2017). Involvement of heparanase in the pathogenesis of acute kidney injury: nephroprotective effect of PG545. Oncotarget.

[29] Zhao H, Alam A, Soo AP, George AJT, Ma D (2018). Ischemia-Reperfusion Injury Reduces Long-Term Renal Graft Survival: Mechanism and Beyond. EBioMedicine.

[30] Norman JT (2006). Protecting the microvasculature: a tight connection to ameliorating chronic kidney disease?. J Am Soc Nephrol.

[31] El-Zoghby ZM, Stegall MD, Lager DJ, Kremers WK, Amer H, Gloor JM, Cosio FG (2009). Identifying specific causes of kidney allograft loss. Am J Transplant.

[32] Li J, Meng X, Hu J, Zhang Y, Dang Y, Wei L, Shi M (2017). Heparanase promotes radiation resistance of cervical cancer by upregulating hypoxia inducible factor 1. Am J Cancer Res.

[33] Nangaku M (2006). Chronic hypoxia and tubulointerstitial injury: a final common pathway to end-stage renal failure. J Am Soc Nephrol.

[34] Zeisberg M, Kalluri R (2008). Fibroblasts emerge via epithelial-mesenchymal transition in chronic kidney fibrosis. Front Biosci.

[35] LeBleu VS, Taduri G, O'Connell J, Teng Y, Cooke VG, Woda C, Sugimoto H, Kalluri R (2013). Origin and function of myofibroblasts in kidney fibrosis. Nat Med.

[36] Lovisa S, Zeisberg M, Kalluri R (2016). Partial Epithelial-to-Mesenchymal Transition and Other New Mechanisms of Kidney Fibrosis. Trends Endocrinol Metab.

[37] Lovisa S, LeBleu VS, Tampe B, Sugimoto H, Vadnagara K, Carstens JL, Wu CC, Hagos Y, Burckhardt BC, Pentcheva-Hoang T, Nischal H, Allison JP, Zeisberg M (2015). Epithelial-to-mesenchymal transition induces cell cycle arrest and parenchymal damage in renal fibrosis. Nat Med.

[38] Grande MT, Sánchez-Laorden B, López-Blau C, De Frutos CA, Boutet A, Arévalo M, Rowe RG, Weiss SJ, López-Novoa JM, Nieto MA (2015). Snail1-induced partial epithelial-to-mesenchymal transition drives renal fibrosis in mice and can be targeted to reverse established disease. Nat Med.

[39] Chetty C, Lakka SS, Bhoopathi P, Rao JS (2010). MMP-2 alters VEGF expression via alphaVbeta3 integrin-mediated PI3K/AKT signaling in A549 lung cancer cells. Int J Cancer.

[40] Mantuano E, Inoue G, Li X, Takahashi K, Gaultier A, Gonias SL, Campana WM (2008). The hemopexin domain of matrix metalloproteinase-9 activates cell signaling and promotes migration of schwann cells by binding to low-density lipoprotein receptor-related protein. J Neurosci.

[41] Zaza G, Masola V, Granata S, Pontrelli P, Sallustio F, Gesualdo L, Gambaro G, Grandaliano G, Lupo A (2014). Dialysis-related transcriptomic profiling: the pivotal role of heparanase. Exp Biol Med (Maywood).

[42] Lv W, Booz GW, Wang Y, Fan F, Roman RJ (2018). Inflammation and renal fibrosis: Recent developments on key signaling molecules as potential therapeutic targets. Eur J Pharmacol.

[43] Kalogeris T, Baines CP, Krenz M, Korthuis RJ (2016). Ischemia/Reperfusion. Compr Physiol.

[44] Secchi MF, Masola V, Zaza G, Lupo A, Gambaro G, Onisto M (2015). Recent data concerning heparanase: focus on fibrosis, inflammation and cancer. Biomol Concepts.

[45] Sureshbabu A, Ryter SW, Choi ME (2015). Oxidative stress and autophagy: crucial modulators of kidney injury. Redox Biol.

[46] Tabriziani H, Lipkowitz MS, Vuong N (2018). Chronic kidney disease, kidney transplantation and oxidative stress: a new look at successful kidney transplantation. Clin Kidney J.

[47] Signorini L, Granata S, Lupo A, Zaza G (2017). Naturally Occurring Compounds: New Potential Weapons against Oxidative Stress in Chronic Kidney Disease. Int J Mol Sci.

[48] Hamoud S, Shekh Muhammad R, Abu-Saleh N, Hassan A, Zohar Y, Hayek T (2017). Heparanase Inhibition Reduces Glucose Levels, Blood Pressure, and Oxidative Stress in Apolipoprotein E Knockout Mice. BioMed Res Int.

[49] Lv W, Booz GW, Fan F, Wang Y, Roman RJ (2018). Oxidative Stress and Renal Fibrosis: Recent Insights for the Development of Novel Therapeutic Strategies. Front Physiol.

[50] Schramm L, La M, Heidbreder E, Hecker M, Beckman JS, Lopau K, Zimmermann J, Rendl J, Reiners C, Winderl S, Wanner C, Schmidt HH (2002). L-arginine deficiency and supplementation in experimental acute renal failure and in human kidney transplantation. Kidney Int.

[51] Foglieni C, Fulgenzi A, Ticozzi P, Pellegatta F, Sciorati C, Belloni D, Ferrero E, Ferrero ME (2006). Protective effect of EDTA preadministration on renal ischemia. BMC Nephrol.

[52] O'Riordan E, Mendelev N, Patschan S, Patschan D, Eskander J, Cohen-Gould L, Chander P, Goligorsky MS (2007). Chronic NOS inhibition actuates endothelial-mesenchymal transformation. Am J Physiol Heart Circ Physiol.

[53] Goligorsky MS, Brodsky SV, Noiri E (2002). Nitric oxide in acute renal failure: NOS versus NOS. Kidney Int.

[54] Schneider R, Raff U, Vornberger N, Schmidt M, Freund R, Reber M, Schramm L, Gambaryan S, Wanner C, Schmidt HH, Galle J (2003). L-Arginine counteracts nitric oxide deficiency and improves the recovery phase of ischemic acute renal failure in rats. Kidney Int.

[55] Yen W, Cai B, Yang J, Zhang L, Zeng M, Tarbell JM, Fu BM (2015). Endothelial surface glycocalyx can regulate flow-induced nitric oxide production in microvessels in vivo. PLoS One.

[56] Garsen M, Rops AL, Li J, van Beneden K, van den Branden C, Berden JH, Rabelink TJ, van der Vlag J (2016). Endothelial Nitric Oxide Synthase Prevents Heparanase Induction and the Development of Proteinuria. PLoS One.

[57] Masola V, Zaza G, Onisto M, Lupo A, Gambaro G (2014). Glycosaminoglycans, proteoglycans and sulodexide and the endothelium: biological roles and pharmacological effects. Int Angiol.

[58] Barton M (2008). Reversal of proteinuric renal disease and the emerging role of endothelin. Nat Clin Pract Nephrol.

[59] Drakopoulos A, Goumenos DS, Vlachojannis JG, Tsakas S (2006). Endothelin receptors in the kidney of patients with proteinuric and non-proteinuric nephropathies. Ren Fail.

[60] Dhaun N, Goddard J, Webb DJ (2006). The endothelin system and its antagonism in chronic kidney disease. J Am Soc Nephrol.

[61] Niu J, Wu J, Li X, Zhang F (2015). Association between endothelin-1/endothelin receptor A and inflammation in mouse kidneys following acute ischemia/reperfusion. Mol Med Rep.

[62] Garsen M, Lenoir O, Rops AL, Dijkman HB, Willemsen B, van Kuppevelt TH, Rabelink TJ, Berden JH, Tharaux PL, van der Vlag J (2016). Endothelin-1 Induces Proteinuria by Heparanase-Mediated Disruption of the Glomerular Glycocalyx. J Am Soc Nephrol.

[63] Stow LR, Jacobs ME, Wingo CS, Cain BD (2011). Endothelin-1 gene regulation. FASEB J.

[64] Farrugia BL, Lord MS, Melrose J, Whitelock JM (2018). The Role of Heparan Sulfate in Inflammation, and the Development of Biomimetics as Anti-Inflammatory Strategies. J Histochem Cytochem.

[65] Shaker RA, Abboud SH, Assad HC, Hadi N (2018). Enoxaparin attenuates doxorubicin induced cardiotoxicity in rats via interfering with oxidative stress, inflammation and apoptosis. BMC Pharmacol Toxicol.

[66] Barbieri P, Paoletti D, Giannini G, Sanderson RD, Noseda A (2017). Roneparstat and Heparanase Inhibition: A New Tool for Cancer Treatment-ment. J Pharmacol Clin Toxicol.

[67] Galli M, Chatterjee M, Grasso M, Specchia G, Magen H, Einsele H, Celeghini I, Barbieri P, Paoletti D, Pace S, Sanderson RD, Rambaldi A, Nagler A (2018). Phase I study of the heparanase inhibitor Roneparstat: an innovative approach for multiple myeloma therapy. Haematologica.

[68] Secchi MF, Crescenzi M, Masola V, Russo FP, Floreani A, Onisto M (2017). Heparanase and macrophage interplay in the onset of liver fibrosis. Sci Rep.

[69] Masola V, Granata S, Bellin G, Gambaro G, Onisto M, Rugiu C, Lupo A, Zaza G (2017). Specific heparanase inhibition reverses glucose-induced mesothelial-to-mesenchymal transition. Nephrol Dial Transplant.

[70] Murawski IJ, Maina RW, Gupta IR (2010). The relationship between nephron number, kidney size and body weight in two inbred mouse strains. Organogenesis.

